# Development and application of a real-time polymerase chain reaction assay to detect lumpy skin disease virus belonging to the Kenyan sheep and goat pox group

**DOI:** 10.1186/s13104-023-06502-z

**Published:** 2023-09-30

**Authors:** Alexander Sprygin, Ali Mazloum, Antoinette Van Schalkwyk, Alena Krotova, Olga Bydovskaya, Larisa Prokhvatilova, Ilya Chvala

**Affiliations:** 1 Federal Center for Animal Health, Vladimir, Russia; 2 Agricultural Research Council-Onderstepoort Veterinary Institute, Onderstepoort, South Africa; 3 Department of Biotechnology, University of the Western Cape, Bellville, South Africa

**Keywords:** Polymerase chain reaction, Kenyan sheep and goat pox, Vaccine, Recombination, Real-time, Epidemiology

## Abstract

Lumpy skin disease (LSD) outbreaks in Southeast and South Asia are attributed to different lineages of LSD virus (LSDV). Variants belonging to the novel recombinant cluster 2.5 circulate in China and Thailand, while a Kenyan sheep and goat pox (KSGP) strain from cluster 1.1 circulates in India, Pakistan, and Bangladesh. The clusters representing these circulating strains are vastly different. However, if their distribution encroaches into each other’s ranges, it will be impossible to differentiate between them due to the lack of suitable molecular tools. Thus, fit-for-purpose molecular tools are in demand to effectively and timeously diagnose and investigate the epidemiology of LSDVs in a region. These could significantly contribute to the phylogenetic delineation of LSDVs and the development of preventive measures against transboundary spillovers. This work aimed to develop a real-time polymerase chain reaction assay targeting open reading frame LW032, capable of specifically detecting KSGP-related isolates and recombinant LSDV strains containing the KSGP backbone. The analytical specificity was proven against the widest possible panel of recombinant vaccine-like LSDV strains known to date. The amplification efficiency was 91.08%, and the assay repeatability had a cycle threshold variation of 0.56–1.1 over five repetitions across three runs. This KSGP-specific assay is reliable and fast and is recommended for use in LSDV epidemiological studies where the accurate detection of KSGP genetic signatures is a priority, particularly in regions where KSGP-like and other lineages are circulating.

## Introduction

Lumpy skin disease (LSD) is a disease caused by the lumpy skin disease virus (LSDV), which belongs to the genus *Capripoxvirus* along with sheep pox virus (SPPV) and goat pox virus (GTPV), which are considered emerging pathogens that pose a significant threat to the global livestock industry [1]. All three diseases are listed as notifiable to the World Organization for Animal Health ( [2]. LSDV primarily affects cattle, while SPPV and GTPV affect sheep and goats, respectively [3]. Similar to other viruses in the Poxviridae family, LSDV has a brick-shaped structure containing a linear double-stranded DNA genome of approximately 151 kilo-base pairs (kbp) in length and encoding 156 open reading frames (ORFs). The genome consists of a central coding region flanked by identical 2.4 kbp-inverted terminal repeat regions [4].

LSD has been reported not only in cattle but also in water buffaloes and game animals, particularly antelopes in sub-Saharan Africa [5–7]. This poses a serious economic threat not only to the global cattle but also to the wildlife industries. This disease was first reported in Zambia in the 1920s and has been confined to the African continent for the majority of the 20th century [8]. However, in the last decade, the virus spread beyond the borders of Africa and was reported in the Middle East, Turkey, and Azerbaijan, reaching the European Union, the Balkans, Russia, and Kazakhstan in 2015. Subsequently, the disease was reported in China, Vietnam, Thailand, India, Bangladesh, and Nepal in 2019 [9–13].

The molecular characterization of the known LSDV isolates has led to their subdivision into nine clusters. Before 2017, the two main clusters were clusters 1.1 (Neethling) and 1.2 (Kenyan sheep and goat pox (KSGP)-like). Since 2017, molecular epidemiology has changed, with the first reports of novel LSDV recombinant vaccine-like variants responsible for outbreaks in Russia between 2017 and 2021 [14]. Since the description of Saratov/Russia/2017 (cluster 2.1) in 2017, additional full genome sequences of novel recombinants have been described, resulting in subclusters 2.1–2.5 [15–17]. The novel lineage first described in China in 2019 (cluster 2.5) is currently the dominant lineage circulating in Southeast Asia [18, 19].

Genetic evidence from the ongoing pandemics in India and Bangladesh revealed that the causative agent belongs to the KSGP-like subgroup within cluster 1.2 [20, 21]. Although the origin of this vaccine strain to the Indian subcontinent and the multiple recombinant strains in Southeast Asia remain elusive, the circulation of these strains raises concerns regarding the diagnostics and epidemiology in the region [22–24]. Currently, assays are available to detect LSDV at the species level and even to differentiate between vaccine and field strains. However, no assay has been reported to detect specifically KSGP-like strains [25, 26].

This study aimed to develop a specific real-time polymerase chain reaction (PCR) assay capable of detecting and differentiating KSGP-like genetic signatures. The sensitivity and specificity of the assay were evaluated using the largest available panel of LSDV strains. The assay was capable of selectively detecting not only KSGP but also recombinant strains containing the KSGP backbone.

## Methods

### Samples and viruses

A panel of 27 unique isolates representing all the identified genome clusters was used to validate the reported real-time PCR assay (Table 1).

**Table 1:** A panel of strains used for the assay specificity validation

No.	Isolate/strain*	Ct	Parental strains	Sample	Ct** (25)	Phylogenetic cluster	Accession number of full genome
1	Dagestan/2015	Neg	Not applicable	Cell culture	15.10	1.2	MH893760
2	Ethiopia/1995	Neg	Not applicable	Cell culture	23.5	1.2	Not available
3	Saratov/2017	Neg	Neethling-major, KSGP-minor	Cell culture	22.30	2.1	MH646674
4	Kurgan/2018	14.89	KSGP-major, Neethling-minor	Skin scabs	13.75	2.6	OP948721
5	Chelyabinsk/2018	Neg	Neethling-major, KSGP-minor	Skin scabs	16.91	Not determined	Not available
6	Samara/2018	21.68	KSGP-major, Neethling-minor	Skin scabs	20.89	Not determined	Not available
7	Udmurtiya/2019	22.23	KSGP-major, Neethling-minor	Cell culture	22.14	2.2	MT134042
8	Saratov/2019	Neg	Neethling-major, KSGP-minor	Cell culture	25.42	2.1	OM530217
9	Tyumen/2019	Neg	Neethling-major, KSGP-minor	Cell culture	23.17	2.4	OL542833
	Khabarovsk/2020	Neg	Neethling-major, KSGP-minor	Cell culture	19.87	2.5	OM793603
10	EAO/2020	Neg	Neethling-major, KSGP-minor	Nasal swab	16.77	2.5	Not available
11	Altay/2020	Neg	Neethling-major, KSGP-minor	Cell culture	23.65	2.5	OP948720
12	Tomsk/2020	Neg	Neethling-major, KSGP-minor	Cell culture	24.41	2.5	OM793602
13	Mongolia/2021	Neg	Neethling-major, KSGP-minor	Skin scab	18.50	2.5	Not available
14	Buryatiya/2021	Neg	Neethling-major, KSGP-minor	Cell culture	20.16	2.5	OP948726
15	Zabaykalsky/2021	Neg	Neethling-major, KSGP-minor	Cell culture	26.20	2.5	OP948719
16	Amur/2022	Neg	Neethling-major, KSGP-minor	Cell culture	26.31	2.5	Not available
17	Buryatiya/2021	Neg	Neethling-major, KSGP-minor	Cell culture	20.16	2.5	OP948726
18	Tuva/2022	Neg	Neethling-major, KSGP-minor	Cell culture	19.25	2.5	Not available
19	KSGPO-240/Kenya/1959	25.17	Not applicable	DNA	24.90	1.2-KSGPO	KX683219
20	Lumpivax vaccine	27.41	Not applicable	DNA	27.62	1.2-KSGPO	Not available
21	Sverdlovsk/2018	31.01	Not determined	Nasal swab	31.22	Not determined	Not available
22	Omsk/2018	29.00	Not determined	Nasal swab	28.64	Not determined	Not available
23	Afghanistan	Neg	Not applicable	Cell culture	Neg	Sheep pox	Not available
24	NISKHI	Neg	Not applicable	Cell culture	Neg	Sheep pox	AY077834
25	Moscow/2018	Neg	Not applicable	Cell culture	Neg	Sheep pox	ON961656
26	Dagestan/2020	Neg	Not applicable	Skin scabs	Neg	Sheep pox	OQ434236
27	Amur/2018	Neg	Not applicable	Skin scabs	Neg	Sheep pox	OQ434235

Ct: cycle threshold

*Samples used in this work are available at the collection of microorganisms at the Federal Center for Animal Health, Vladimir, Russia

**Ct results based on the real-time PCR screening assay for the universal detection of lumpy skin disease virus DNA developed earlier in 2019 [25]

### DNA extraction

Viral genomic DNA was extracted using the phenol–chloroform extraction protocol as described previously [27].

### Primer design

**Fig. 1:**
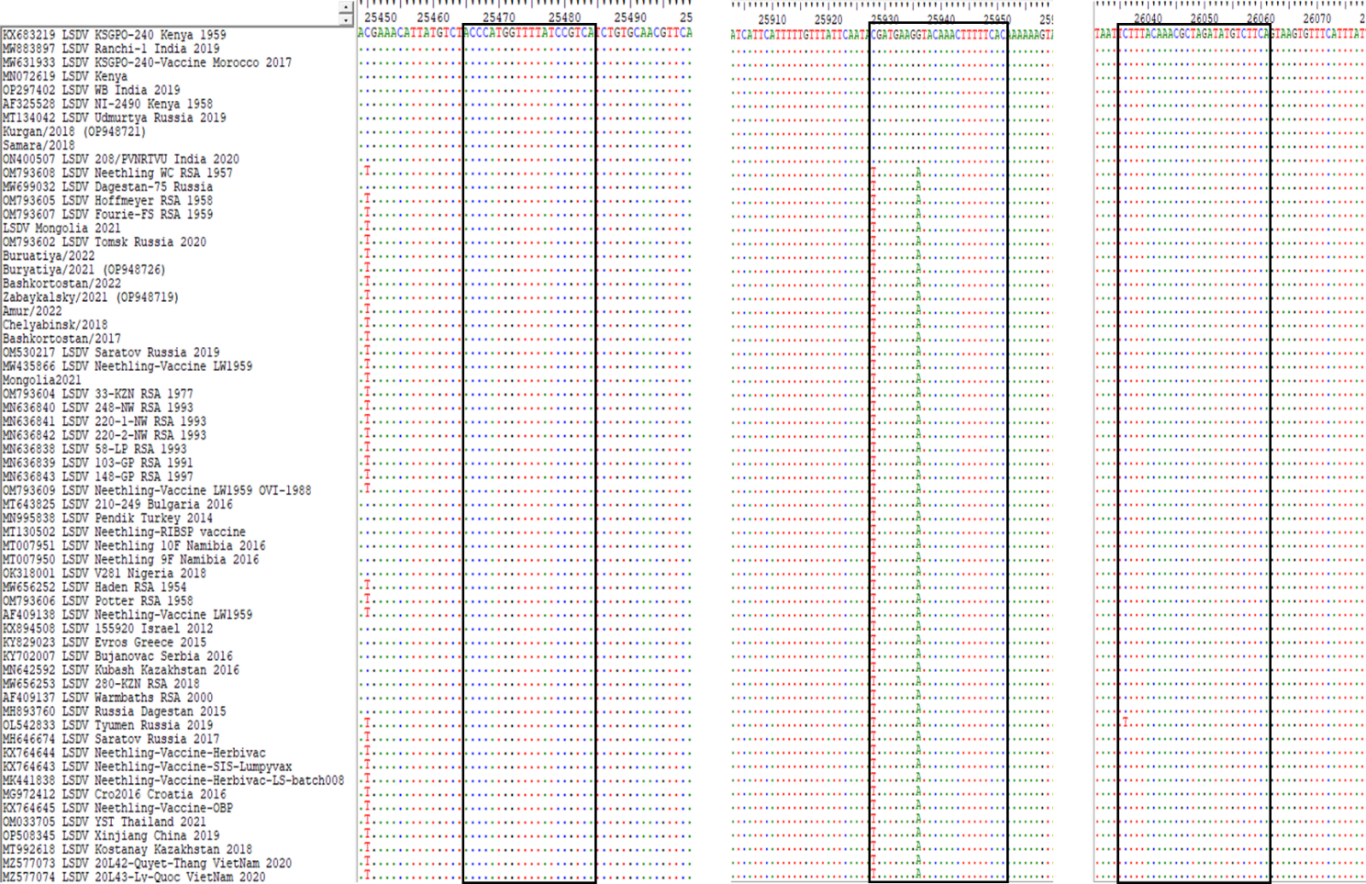
An alignment of the primers and probe-binding region, containing all the available LSDV strains, representing the seven different clusters

The poly(A) polymerase catalytic subunit encoded by ORF LW032 was selected since it is conserved across the available sequences. No recombination event has yet been observed in this locus. Subsequently, the primers F 5-ACCCATGGTTTTATCCGTCA-3 and 5-TGAAGACATATCTAGCGTTTGTAAAGA-3 were designed to amplify a 610 bp region, while the probe FAM 5-[C-lna]GATGAAG[G-lna]TACAAACTTTTTCAC-3 BHQ-1 selectively annealed to KSGP-like strains. The alignment of the binding region of the probe is shown in Fig. 1.

### PCR protocol

PCR reaction setup, protocol, and statistical analysis where performed as previously described [28, 29]. We focused on the minimal critical parameters needed to ascertain the assay reliability: analytical specificity, analytical sensitivity (limit of detection), reaction efficiency, repeatability.

### Analytical specificity

The specific detection of KSGP-like signatures was verified using DNA containing individual strain nucleic acids and a mixture of strain DNA mimicking the co-infection. All strains used for validation had a Ct value not higher than 30 to ensure the specificity. The assay did not produce any false positives when tested against sheep pox virus with a high titer (skin scabs and cell culture with an average Ct value of 13.11–17.12). Cross-reactivity against sheep pox virus is chosen due to the high genetic similarity across the Capripoxvirus genus [4].

### Analytical sensitivity

The limit of detection (LOD) of the PCR assay was determined using a serial dilution of different LSDV genomic DNA, starting with the Udmurtiya/2019 virus strain containing a titer of 5.75 lg TCD50/cm^3^. LOD was defined as at least 95% positive replicates at the terminal dilution of 20 replicates tested [28]. Five tenfold dilutions were initially prepared, followed by three twofold serial dilutions. The reaction efficiency was determined from the slope using the following equation:

*E = [10 (slope) − 1] × 100*,

where *E* is the reaction efficiency, and *E* = 100 corresponds to 100% efficiency. The repeatability and coefficient of variation (CV) were assessed by examining the same five tenfold dilutions (Udmurtiya/2019, with a starting titer of 5.75 lg TCD50/cm^3^) in five repetitions on three different days. Statistic evaluation was performed using Statistica v.10 (StatSoft, Tulsa, OK, USA).

## Results

Before evaluating the analytical characteristics of the assay, the specificity was first examined against a wide collection of naturally occurring recombinant vaccine-like strains and other capripoxviruses (Table 1). This is the most diverse panel of LSDV currently described, providing a unique opportunity to validate diagnostic tools for LSDV research. Since the probe annealing region differs by a few nucleotides between KSGP and non-KSGP strains, the probe contained locked nucleic acid bases to enhance the binding specificity. Therefore, the assay demonstrated specific detection of only target LSDV sequences. The latter is specific to the original KSGP virus DNA or DNA of recombinant strains whose major parental strain is KSGP (Table 1) [30]. No cross-reaction with related LSDV DNA or nontarget SPPV samples was detected (Table 1). Since KSGP-like strains of Indian origin were unavailable for the study, considering the conservative nature of poxvirus genomes [17], the assay is confidently deemed readily specific toward Indian KSGP.

**Fig. 2:**
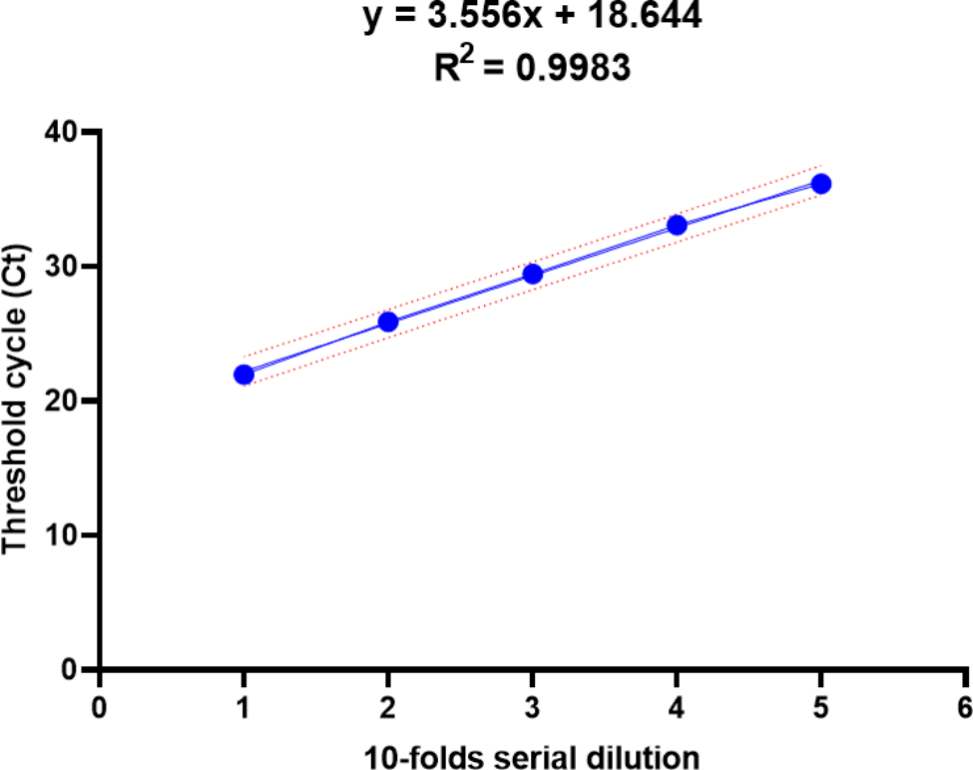
Linear regression constructed over serial 10-fold dilutions. The equation of the standard curve obtained was *y* = 3.556x + 18.644, and the coefficient of determination (R^2^) was 0.9983

The amplification efficiency over five orders of magnitude was 91.08%, with the variation ranging from 0.54 to 1.18 (Fig. 2).

The repeatability of the assay was assessed across three replicates, calculated by the percentage of total variance obtained from five repetitions of a single sample in one run. A low variation in CV was obtained: the SD and CV ranged from 0.56 to 1.1 and 1.87–3.64%, respectively, over five repetitions across three runs (Table 2). As for LOD, the assay sensitivity was found to be the fifth tenfold dilution with a titer of 0.75 lg TCD50/cm^3^.

**Table 2:** Repeatability of the assay in one run (5 repetitions) and across 3 runs (15 repetitions)

PCR	Mean Ct	SD	СV (%)
Run 1	29.68	0.97	3.27
Run 2	30.26	1.1	3.64
Run 3	29.93	0.56	1.87
*n* = 15			
Across runs	29.96	0.88	2.94

PCR: polymerase chain reaction; Ct: cycle threshold; SD: standard deviation; CV: coefficient of variation

## Discussion

LSD can be successfully controlled by proper diagnostic tools [31]. Recently, a few lineage-specific assays have been reported. Along with the pan LSDV assay by Sprygin [25], the PCR assay as a DIVA (Differentiating Infected from Vaccinated Animals) strategy has also been developed and validated on clinical samples [32–34]. The ORF LW008 vaccine assay can detect Neethling vaccine DNA and DNA of some recombinant vaccine-like strains [31, 35], whereas the GPCR vaccine assay targets Neethling DNA, KSGP DNA, and DNA of some recombinant vaccine-like strains, which significantly limits the application of these assays in regions where recombinant vaccine-like LSDV is present [36]. Haegeman A. et al., 2023 published the first manuscript on development and validation of a new DIVA qPCR for differentiation between the Neethling vaccine strain from the LSDV recombinant strains currently circulating in Asia [37]. With the emergence of recombinant vaccine-like strains of LSDV in Southeast Asia coupled with one more pandemic with another lineage within cluster 1.2 (KSGP) in India and Bangladesh, it is crucial to have reliable and thoroughly validated laboratory instruments for timely detection and diagnosis [38]. The protocol developed by Haegeman A. et al., is capable on identifying recombinant isolates that have Neethling genome as a major parental backbone, but doesn’t fit for recombinant isolates with KSGP-vaccine genome as a major parental backbone.

While the risks of mutual spillovers are high in countries located at the interface of affected regions, followed by coinfection of animals, the lack of instruments to differentiate between cluster 2.5 and cluster 1.2 KSGP subcluster would have a profound influence on the understanding and control of LSD in Southeast and South Asia [17]. Interestingly, the Kenyan strain lineage was restricted to some African regions in the past, so its molecular detection was not the priority, and then it fell within the field cluster 1.2 [18]. However, a recent resurgence of recombinant LSDV whose parental strain is KSGP and the concomitant rise of KSGP-related outbreaks in South Asia showed an objective need for such tools [21]. In this study, a specific PCR assay capable of detecting KSGP genetic signatures was developed (Tables 1 and 2). Notably, the assay was proved to be KSGP-specific regardless of whether it is a recombinant strain with the KSGP backbone, such as Kurgan/2018 or Udmurtiya/2019, or the original KSGP strain (Table 2). Unfortunately, KSGP-like strains of Indian origin were unavailable for the study. However, considering the conservative nature of poxvirus genomes [39], this assay will readily differentiate Indian LSDV strains as well. The performance characteristics, such as analytical sensitivity and amplification efficiency, were validated according to recommended Minimum Information for Publication of Quantitative Real-Time PCR Experiments (MIQE) [28], OIE Validation Guideline [29] and compared well with reported assays.

This KSGP assay opens up new possibilities in molecular diagnostics. In combination with the ORF126 assay that targets isolates from cluster 1.2, the differentiation of field isolates into subclusters is now accessible: one including Dagestan/2015, Serbia, Warmbaths, and Israel [17] and the other including KSGP, Indian LSDV [20], Bangladesh LSDV [21], and recombinants (Kurgan/2018, Samara/2018, and Udmurtiya/2019) (Table 2). As a complementary add-on to this tool, since the Kenyan Lumpivax contains KSGP, this tool can be used for DIVA if this KSGP-based vaccine is administered.

In this work, we developed a novel PCR assay against a panel of unique LSDV strains available in FGBI ARIAH (Vladimir, Russia), that can specifically detect KSGP-like genetic signatures. Not only outbreaks can be investigated, but a DIVA approach (differentiation infected animals from vaccinated animals) can also be implemented with particular regard to the Kenyan vaccine (KSGP strain), the use of which precipitated the emergence of all currently known recombinant stains [17]. This new tool will significantly improve the molecular epidemiological studies tracking the ongoing spread of different LSDV lineages, which is essential for the understanding of LSDV transmission throughout affected regions and in tandem with the assay by Haegeman et al. (2023) more detailed information on the molecular epidemiology of LSDV worldwide will be gained. Overall, this assay holds promise and is recommended for use in countries where the risks of KSGP-related outbreaks can occur or where the KSGP based vaccine is in use. In the future, this assay will be validated and tested on a larger collection of samples to estimate such parameters as the diagnostic sensitivity and diagnostic specificity, where WOAH guidelines 3.2.6 will be considered as reference for research methodology [29].

### Limitations

This assay demonstrated good specificity based on the available samples. Samples of LSDV DNA from India and Bangladesh, where the target lineage is circulating also, would complement the specificity testing and guarantee that the proposed assay perfectly fit for purpose.

## Data Availability

All data are presented within the manuscript.

## Abbreviations

LSD: Lumpy skin disease
LSDV: LSD virus
KSGP: Kenyan sheep and goat pox
SPPV: sheep pox virus
GTPV: goat pox virus
kbp: kilobase pairs
ORF: open reading frame
LOD: limit of detection
CV: coefficient of variation (CV)
TCD50: 50% Tissue Culture Infectious Dose
R^2^: coefficient of determination
DIVA: Differentiating Infected from Vaccinated Animals
BHQ-1: Black Hole Quencher-1
Ct: threshold cycle
FAM: 6-carboxy fluorescein
LNA: Locked Nucleic Acid
